# The Meis homeoprotein regulates the axolotl Prod 1 promoter during limb regeneration

**DOI:** 10.1016/j.gene.2011.06.003

**Published:** 2011-09-15

**Authors:** Nooreen Shaikh, Phillip B. Gates, Jeremy P. Brockes

**Affiliations:** Institute of Structural and Molecular Biology, University College London, Gower Street, London, England WC1E 6BT, UK

**Keywords:** RA, retinoic acid, PD, proximodistal, dpa, days post amputation, Salamander, Proximodistal, Blastema

## Abstract

During limb regeneration in salamanders the blastemal cells give rise only to structures distal to the level of amputation. This proximodistal identity can be regulated by ectopic expression of Meis homeoproteins or the three finger protein Prod 1 which acts at the cell surface. It has been suggested that Meis acts by regulating the transcription of Prod 1. We have sequenced the axolotl Prod 1 promoter and selected two candidate sites for binding Meis homeoproteins. The sites were mutated in various combinations in promoters expressing a luciferase reporter gene. The promoter activity was assayed by nucleofecting AL1 cells, a cultured axolotl limb cell line that expresses both Prod 1 and Meis 1 and 2. The activity of the promoter was inhibited by 60% after mutation at Meis site 1, but not at Meis site 2. The promoter constructs were electroporated into axolotl limb blastemas and the wild type promoter was more active in a proximal blastema than in a contralateral distal blastema. The wild type promoter was significantly more active than a (site 1 + site 2) mutant promoter in contralateral proximal blastemas, but the promoters were equivalent in contralateral distal blastemas. The separate site 1 or site 2 mutants were not significantly different from wild type in contralateral proximal blastemas, thus contrasting with the site 1 results in AL1 cells. These data provide strong support for the hypotheses that the Prod 1 promoter is regulated on the proximodistal axis, and that Meis homeoproteins directly regulate the promoter on this axis during limb regeneration in addition to cultured cells.

## Graphical abstract

**Figure:**
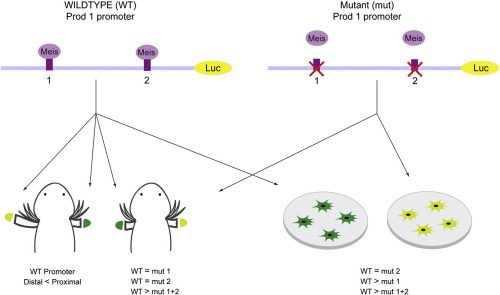

**Research highlights:**

►The Prod 1 promoter is regulated in cultured limb cells by binding of Meis proteins. ►The promoter has higher activity in a proximal regeneration blastema than a distal. ►This PD difference in limb regeneration depends on two Meis sites in the promoter.

## 1 Introduction

Limb regeneration in salamanders proceeds by formation of the blastema, a mound of mesenchymal progenitors at the end of the stump (Iten and Bryant, 1973; Kragl et al., 2009). The blastemal cells derive important aspects of their identity not only from their cell type of origin but also from the position of the amputation plane on the proximodistal (PD) axis that extends from shoulder to finger tip. A blastema always gives rise to structures distal to its level of origin except after exposure to retinoic acid (RA) or precursor retinoids (Maden, 1982; Thoms and Stocum, 1984). This leads to a dose-dependent respecification of distal cells to a more proximal identity. The molecular basis for PD identity is a subject of much current interest both in relation to limb regeneration and limb development. There are several assays for the cellular basis of PD identity in regeneration that reflect the consequences of apposing proximal and distal blastemas, and these include engulfment, intercalation and affinophoresis (Crawford and Stocum, 1988; Nardi and Stocum, 1983; Pescitelli and Stocum, 1980). These assays have suggested that one aspect of this identity may be expressed at the cell surface so as to mediate cell–cell interactions. This led to the identification from a differential screen of the newt three finger protein called Prod 1(da Silva et al., 2002). Prod 1 is up-regulated by RA, is expressed at higher levels in proximal limb cells, and is located at the cell surface with a GPI glycolipid anchor (da Silva et al., 2002; Kumar et al., 2007a). Antibodies to Prod 1 specifically block the process of PD engulfment in culture (da Silva et al., 2002). Interestingly axolotl Prod 1 is a secreted protein that is functionally equivalent to newt Prod 1, and both proteins apparently signal to the cell by interaction with the EGF receptor (2011). The distinctive alpha-helical region of Prod 1 has recently been shown to be critical for its signalling activity, and also for the ability to function in both anchored and anchorless versions (Blassberg et al., 2011).

In the context of limb development there has been considerable progress in relation to the transcriptional regulation of PD identity. The Meis genes encode transcription factors of the TALE class of homeoproteins, and are activated by RA during patterning of the PD axis in limb development (Mercader et al., 2000; Yashiro et al., 2004). The ectopic overexpression of Meis 1 in distal cells of the chick and mouse limb bud leads to a proximal shift of identity along the PD axis (Mercader et al., 1999). The Meis proteins may interact with paired box proteins (PBX), a second class of the TALE family, although evidence has been presented that this interaction is not required for PD specification in mouse limb development (Mercader et al., 2009). Various target sites have been identified for Meis and PBX-Meis proteins. The consensus Meis-binding sequence is usually written as TGACAG/A (Chang et al., 1997), but the site is active in the reverse orientation and was identified as the reverse complement TTGTCA in a 26 base pair element within the mouse and chicken Pax 6 regulatory sequences (Zhang et al., 2002). The PBX site consensus TGAT can occur in conjunction with the Meis site with variable spacing and orientation and bind to PBX-Meis heterodimers as demonstrated for the mouse HoxB2 enhancer (Jacobs et al., 1999).

The expression of Meis 1 and 2 genes is higher in a proximal axolotl limb blastema than a distal one and RA treatment up regulates their expression in a distal blastema (Mercader et al., 2005). The downstream targets of Meis action that mediate PD identity are unknown in any context. It is possible that there is a regulatory interaction between the transcription factor Meis and the promoter of Prod 1, a protein acting at the cell surface. The strongest evidence to date is that they share the same distinctive phenotype after overexpression in the axolotl limb blastema. If distal blastemal cells are electroporated with a plasmid expressing newt or axolotl Prod 1, or axolotl Meis 1 and 2, they relocate to a more proximal location in the regenerate and contribute to proximal structures (Echeverri and Tanaka, 2005; Mercader et al., 2005). The present study tests the hypothesis that Meis directly regulates the expression of the Prod 1 gene. We have isolated the axolotl Prod 1 promoter and shown that Meis binding sites regulate its activity in cultured cells and in proximal blastemal cells in vivo. This work raises several issues for further understanding of PD identity in regeneration, and serves also to establish the feasibility of promoter analysis during limb regeneration.

## 2 Materials and methods

### 2.1 Animal husbandry

Axolotls (Ambystoma mexicanum) were obtained from Neil Hardy Aquatica (Croydon, UK) and maintained in individual aquaria between 14 and 18 °C. Larvae of 4–6 cm size were anesthetised in 0.1% tricaine prior to amputation at the mid-humerus for proximal blastemas, and at the distal radius/ulna for distal blastemas.

### 2.2 Transfection of amphibian and mammalian cells

AL1 cells were obtained from S.Roy (University of Montreal) and cultured as described (Roy et al., 2000; Villiard et al., 2007). Cos 7 cells were obtained from ATCC, and cultured by standard methods. They were transfected using Lipofectamine 2000 (Invitrogen) according to the manufacturer's instructions. AL1 cells were transfected by nucleofection using the Lonza nucleofection apparatus and reagents. The cell suspension (10^5^ cells in 0.1 ml) was mixed with 2 μg firefly luciferase DNA and 1 μg Renilla DNA, and nucleofected as according to manufacturer's instructions. The nucleofected cells were added to 1.5 ml supplemented minimal medium (MEM, Gibco) and incubated at 25 °C prior to plating into gelatin-coated dishes.

Cos 7 cells were lysed at 24 h post-transfection, and AL1 cells at 48 h, both using the passive lysis buffer provided in the Dual-luciferase reporter assay kit following the Manufacturer's instructions (Promega). The lysates were subjected to a freeze–thaw cycle, centrifuged to remove debris, and assayed according to the DLR^TM^ assay. Assays were performed in triplicate and read in 96 well plates using a Pherastar detector. The activity of each Prod 1 promoter construct was normalised to the activity of the internal Renilla control and expressed as the ratio (Firefly/Renilla).

### 2.3 Electroporation

Animals were anesthetised at 10 days post amputation in 0.1% tricaine, placed under a stereo microscope (Nikon SMZ800) for electroporation and injected with 5 μg Firefly luciferase plasmid and 1 μg Renilla plasmid into the limb blastema, using a customised micromanipulator connected to a Picospritzer. Ten external pulses (300 V/cm) were applied with customised tweezer electrodes to electroporate the DNA as described (Kumar et al., 2007b). Blastemas were analysed essentially as described above for luciferase activity at 96 h post-electroporation after lysis by homogenisation in 50 μl lysis buffer. It should be noted that there is significant activity at distal levels, this allowing the determination of normalised activity values. For analysis of RFP expression, blastemas were sectioned as described (Kumar et al., 2007b), and examined in a Zeiss Axioskop 2 fluorescence microscope, photographed with a Hamamatsu Orca digital camera, and analysed with Openlab (Perkin Elmer) software.

### 2.4 Recombinant methodology

Genomic DNA was prepared from the fore limbs of 7 axolotls (DNeasy kit, Qiagen) and from this the Axolotl Prod1 promoter was isolated using the universal Genome Walker kit (Clontech). This involves generating pools of adaptor ligated genomic DNA fragments followed by PCR with adaptor specific primers and gene-specific primers. Amplification of the PvuII digested DNA with the primary gene-specific oligo gaaacagggaggcgccgacgagcttcat and the secondary gene-specific oligo gagcttcatgcctgtggccgtccagtca with appropriate adaptor primers gave a 2.1 Kb product which was blunt end cloned into the SmaI site of Bluescribe (Stratagene) and sequenced. The sequence of the axolotl promoter region is available in GenBank as Axolotl Prod 1 [GenBank:HQ873488].

A 1.9 Kb promoter fragment was subcloned into the BamHI and SacI sites of the pGL3 basic vector. Point mutations were introduced into this fragment using the QuickChange mutagenesis kit (Stratagene) and the following oligonucleotides. Meis 315 gaggttcgccgtagcaagtgaatcaacaca and ctgtgttgsttcacttgctacgagcgaacctc. Meis 1324 caggccatgggctagcaacataacaatg and cattgttatgttgctagcccatggcctg. Pbx315 cgtgccaagtgaaaaaacacaggctgatg and catcagcctgtgttttttcacttggcacg.

RNA was isolated from mid-bud blastemas (5 cm larvae) and subconfluent AL1 tissue culture cells using Tri Reagent (Sigma) and random primed cDNA synthesised using Superscript II (Invitrogen). Gene expression was assayed by quantitative real time PCR with iQ SYBR Green supermix (Bio-rad) on a chromo 4 instrument running Opticon 3 software (Bio-rad). All samples were normalised with EF1-α oligos aacatcgtggtcatcggccat and ggaggtgccagtgatcatgtt. Meis 1 oligos were atgccaggggattacgtctcg and cagtagaccacataatttcctgtg. Meis 2 oligos were cgaggcattttccccaaagtag and ctgctgaccatccaatacaaagc. Prod 1 oligos were ggtggcagtgagcacagggt and tggcattcctgtatcagagt. All reactions were run in triplicate and 4 independent RNA preparations were analysed for each sample.

### 2.5 Western blots and band shift assays

Cos 7 cells were grown to 90% confluence in a 10 cm dish and transfected with either Axolotl Meis 1 or control GFP plasmids, using Lipofectamine 2000 reagent as above. At 24 h post-transfection the cells were lysed by freeze/thaw in 0.02 M Hepes (pH 7.9), 0.2 mM EDTA, 1.5 mM MgCl_2_, 0.42 M NaCl, 25% glycerol and centrifuged. The supernatants (5 ug protein) were analysed by SDS polyacrylamide gel electrophoresis, prior to transfer to nitrocellulose and reaction with antibody to mouse Meis homeoprotein (Millipore SC05-779), followed by IR labelled secondary antibodies (Licor) and analysis on the Odyssey imager.

For band shift assays the optimal binding conditions were determined according to the Odyssey infrared EMSA kit (Licor). Binding assays were incubated for 30 min at room temperature with 5 ug cell extract, 50 ug/ml poly dI/dC and 2.5 nM IRDye-labelled oligonucleotide (Thermo scientific) in 10 mM Tris (pH7.5), 50 mM KCl, 3.5 mM DTT, 0.25% Tween-20. DNA-protein complexes were separated on a denaturing 4% polyacrylamide gel in TBE buffer (50 mM Tris, 0.04 mM Boric acid and 0.5 mM EDTA) and analysed on the Odyssey imager at 700 nm. The sequences for the wild type and mutant oligonucleotides were CATTGTTATGTTGTCAGCCCATGGCCTG and CATTGTTATGTTGCTAGCCCATGGCCTG respectively.

### 2.6 Statistical analysis

A ratio *t* test was performed on the log_2_ transformed ratios to test whether the mutant and wild type promoters are equivalent. The ratio *t* test was performed as described in the Prism 4 statistics guide and the p values determined (Motulsky, 2003).

## 3 Results

### 3.1 Isolation and characterisation of the axolotl Prod 1 promoter

**Fig. 1:**
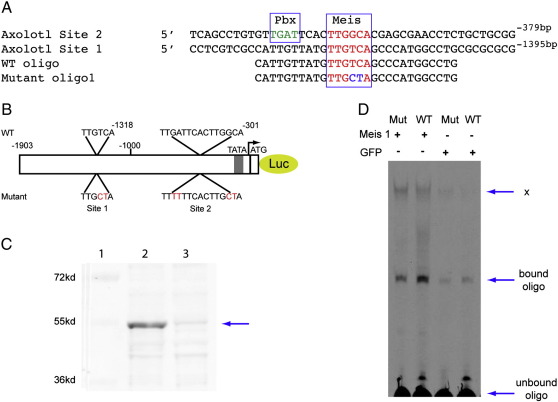
Analysis of Meis sites in the axolotl Prod 1 promoter. (A) The two Meis sites are shown as site 1, a binding site for Meis alone, and site 2, a joint PBX-Meis site. The wild type (WT) and mutated oligonucleotides that were used in the band shift assay in (d) are also shown. (B) Location of the sites in the 1.9 kb sequence, with the mutated versions below. The promoter is shown upstream of a luciferase reporter. (C) Western blot analysis of extracts of transfected Cos 7 cells identifies a band (arrowed) corresponding to axolotl Meis protein. The possibility that this is a target of Meis regulation, rather than Meis itself, cannot be ruled out. Lane 1, Odyssey standard proteins; lane 2, extract of Cos cells transfected with Meis 1 plasmid; lane 3, extract of Cos 7 cells transfected with GFP plasmid as control. Note the immunoreactive band in lane 2, also that the non-specific bands are of comparable intensity. (D) Comparison of wild type and mutant fluorescent oligonucleotides in a Meis 1 bandshift assay. Mutant (mut) or wild type was incubated with extracts of Meis 1 or GFP-transfected Cos 7 cells, and the DNA-protein complexes were separated on a polyacrylamide gel. The band corresponding to free oligonucleotide is shown and a non-specific band X is seen at the top. The putative Meis 1-oligo complex in the middle is of highest intensity for Meis 1-transfected extract interacting with WT oligo (lane 2). This experiment was repeated three times with comparable results. Attempts to demonstrate supershifting of this band with the antibody used in (C) were not successful.

The DNA sequence at a location 5-prime to the transcription start site of axolotl Prod 1 was determined as described in the Methods section. In 1.9 Kb of sequence (Supplementary Information Fig. 1), the hexanucleotide TTGTCA was identified at − 1318 relative to the putative transcription start site and was named site 1. A second TTGGCA was identified downstream of a TGAT putative PBX sequence with 4 base pairs between them. The compound site was named site 2 (Figs. 1A, B).

A double mutation was introduced into positions 4 and 5 of the Meis sequence in sites 1 and 2, and into positions 2 and 3 of the PBX sequence in site 2, as detailed in Fig. 1B. The axolotl Meis protein was expressed by transfection of Cos 7 cells and was detected as a band of 55 kDa in cell lysates after Western blotting with an antibody to Meis. (Fig. 1C, lane 2). In order to evaluate the consequences of the double mutation in the Meis sequence we obtained fluorescent-labelled oligonucleotides containing either the wild type or mutated sequences (Fig. 1A). The labelled wild type and mutated oligonucleotides were incubated with equivalent volumes of Meis-transfected or control cell lysates and analysed on polyacrylamide gel electrophoresis. In Fig. 1D the Meis-shifted oligonucleotide is shown in the middle of the gel while a non-specific shifted band X is shown at the top, and unbound oligonucleotides at the bottom. The Meis protein had a higher affinity for the wild type oligonucleotide as compared to the mutated (Fig. 1d, lanes 1 and 2), indicating the efficacy of the double mutation.

### 3.2 Promoter activity in AL1 cells

**Fig. 2:**
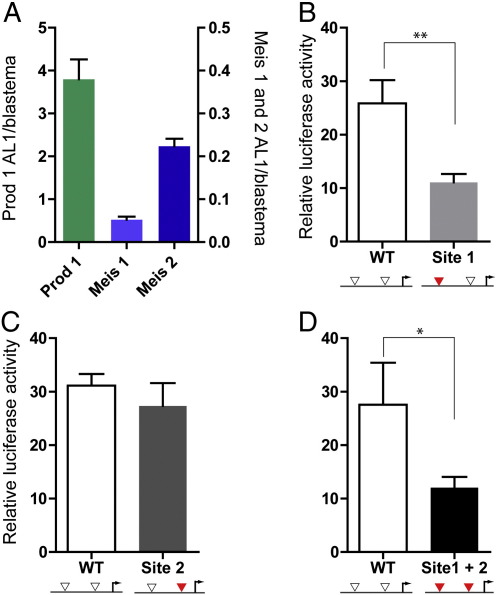
Analysis of Prod 1 promoters by transfection into AL1 cells. (A) Expression levels of Prod 1, Meis 1 and Meis 2 determined by RT-PCR and expressed relative to levels in axolotl limb blastema as described in the Materials and methods section. Note the right hand axis for Meis 1 and 2, and the left hand one for Prod 1. (B) Activity of the wild type and site 1 mutant promoters assayed in parallel. AL1 cells were nucleofected with luciferase plasmids (see Materials and methods section) and lysed 48 h later, prior to determination of the normalised luciferase activity (both promoters, n = 15 transfections; mean and standard error). (C) As for B except that the site 2 mutant promoter was used in place of the site 1 mutant (both promoters, n = 15). D) As for B except the site 1 + site 2 mutant promoter was used in place of the site 1 mutant (both promoters, n = 7). By paired *t* test analyses *P < 0.05; **P < 0.01.

AL1 cells are a cultured cell line originally derived from axolotl limb dermal fibroblasts (Roy et al., 2000; Villiard et al., 2007). The line expresses Prod 1 as well as Meis 1 and Meis 2 as assayed by RT-PCR, although Meis 1 is expressed at significantly lower levels than the limb blastema (Fig. 2A). These cells are efficiently transfected by nucleofection (see Materials and methods section) when over half of the cells express a marker plasmid for red fluorescent protein. The activity of the wild type Prod 1 promoter was analysed by measuring the activity of a Firefly luciferase reporter along with Renilla luciferase expressed from a separate plasmid as a transfection control. The normalised activity of the promoter mutated in the Meis site 1 was assayed in a parallel set of transfections and had only 40% of the wild type activity (Fig. 2B). An independent set of transfections comparing wild type to the site 2 mutant showed no significant difference between the two promoters (Fig. 2C), while the promoter mutated in both sites 1 and 2 had comparable activity to the site 1 mutant (Fig. 2D). These results suggest that the single Meis site at − 1318 is a major determinant for promoter activity and is responsible for 60% of the activity after transfection into AL1 cells.

### 3.3 Electroporation of promoter constructs into the limb blastema

**Fig. 3:**
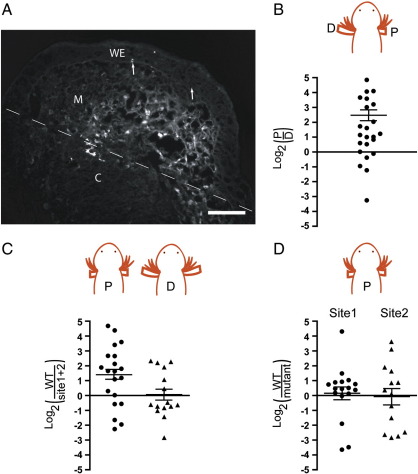
Analysis of Prod 1 promoters after electroporation into the limb blastema. (A) Expression of RFP in a section of a limb blastema. The axolotl limb blastema was electroporated with plasmid DNA expressing RFP and analysed by sectioning as described in the Materials and methods section. The amputation plane is shown with a dotted line and the boundary between the mesenchymal blastema (M) and the wound epithelium (WE) is arrowed. C, cartilage in the limb stump. Scale bar = 40 μm (B) Comparison of Prod 1 promoter activity in contralateral proximal and distal blastemas. The luciferase reporter construct along with the normalising Renilla luciferase plasmid were electroporated into the blastemas of larvae with one proximal and one distal blastema as shown. The ratio of the normalised luciferase activity was plotted for each animal (n = 23) on a log_2_ scale as discussed in the text. The mean and standard error are plotted. (C) Comparison of Prod 1 promoter with site 1 + site 2 mutant promoter after electroporation into contralateral limb blastemas of larvae with proximal (left, n = 20) and distal (right, n = 15) blastemas. (D) Comparison of Prod 1 promoter activity with either site 1 (n = 19) or site 2 (n = 15) mutant promoters after electroporation into contralateral limb blastemas of larvae after proximal amputation.

The axolotl limb blastema can be effectively electroporated with plasmid DNA as shown previously (Echeverri and Tanaka, 2005). A section of a blastema that had been electroporated with a RFP plasmid at 10 dpa and analysed 4 days later is shown in Fig. 3A. We observed that there is little or no labelling of the epidermis or dermis after this procedure whereas the mesenchymal blastema is transfected significantly (Fig. 3A). It is possible that the dense matrix of the dermis does not allow the DNA to enter. This selectivity for the blastemal mesenchyme is clearly advantageous for the interpretation of the present experiments since this compartment expresses PD identity.

A group of larvae were amputated on one side at a proximal level (right side in Fig. 3B), and on the contralateral side at a distal level. The wild type promoter driving Firefly luciferase and a control Renilla luciferase plasmid were electroporated on both sides. After 4 days the blastemas were harvested and extracted, followed by measurement of the dual luciferase activities. The normalised luciferase activity was determined for each side and a ratio calculated for proximal to distal for each animal. There is significant variation between animals in such experiments and in some animals the ratio was less than unity. The data were therefore plotted on a log_2_ scale in order to avoid condensing the reversed ratio points between 0 and 1 on a linear scale. For the 23 animals analysed in Fig. 3B, most (17) show a greater activity in the proximal blastema and the average overall is 6-fold (log_2_ = 2.6). The promoter is apparently more active in a proximal blastema, and this is consistent with greater Prod 1 expression at proximal levels (da Silva et al., 2002; Kumar et al., 2007a).

In a second series of experiments, axolotls were amputated bilaterally at either P or D levels, and electroporated on one side with the wild type promoter luciferase construct and on the other with the (site 1 + site 2) mutant promoter in order to determine if the activity depends on the Meis binding sites. The normalised luciferase activity was determined as before and the ratio of wild type to mutant determined for each animal and plotted on a log_2_ scale (Fig. 3C). The mean ratio for the proximal group is 2.5 (log_2_ = 1.3) and for the distal group is 1.04 (log_2_ = 0.05). A ratio *t* test (Motulsky, 2003) gives a 1% probability that the two promoters are equivalent in activity at a proximal location, and 97% at a distal location. This supports the hypothesis that Meis is a major determinant of Prod 1 promoter activity in a proximal blastema.

The relative activity of wild type and mutant promoters in a proximal blastema was pursued in relation to the site 1 and site 2 mutants in order to determine if the activity depends on one or the other site. For the site 1 mutant the mean value of the ratio was 1.1 (Fig. 3D), and there was no significant difference between wild type and mutant (p = 0.95). For the site 2 mutant the ratio was unity with a p value of 0.95 (Fig. 3D). Thus in contrast to the experiments with AL1 cells in culture (Fig. 2B), either site on its own is responsible for a significant fraction of the promoter activity, and both need to be mutated to knock out full activation in a proximal blastema.

## 4 Discussion

This is the first direct analysis of promoter sequences in salamander regeneration and the electroporation of reporter constructs into the limb blastema has established the validity of this approach. In view of the significant variability of expression within a group of axolotls of the same stage and developmental history, it was necessary in the case of this promoter to consider the results from such groups of 10–20 animals in order to obtain statistical significance. The AL1 cells express Prod 1, and the activity of Meis at site 1 makes a major contribution to the activity of the transfected promoter. The level of the limb from which AL1 cells were derived is not known and this might have affected their properties. In the proximal blastema either site is apparently active and this may reflect the expression of higher levels of Meis 1 and 2 than are present in AL1 cells (Fig. 3A), or possibly the activity of accessory proteins present in one context and not the other. The difference in activity of the wild type and sites 1 and 2 mutant promoters in a proximal blastema is not as great as the difference in the wild type promoter at proximal versus distal locations. This most likely reflects the fact that the mutations did not entirely remove Meis binding activity, or that there are other proteins that are regulating the promoter on the PD axis. Furthermore we have subsequently identified three other potential Meis sites in the promoter and these will need to be analysed by the approaches described here. Nonetheless the present analysis has provided evidence that Prod 1 expression is a target of regulation for Meis homeoproteins acting through the consensus sequences. Our results support the hypothesis that the displacement of distal axolotl blastemal cells after Meis overexpression, as described in the Introduction section, is a consequence of raising the level of Prod 1 expression by direct regulation of its promoter. It has yet to be demonstrated in this context that Prod 1 is upregulated in the electroporated cells.

One important problem in limb regeneration is to understand how the blastemal cells at any PD level derive their particular identity after amputation (Kragl et al., 2009). The most obvious interpretation of the present results would be that Meis is expressed upstream of Prod 1 and that, as proposed for limb development, it is a major determinant of PD identity. In apparent contradiction to this view, Meis is not expressed at higher levels proximally in either a larval axolotl or adult newt intact limb whereas Prod 1 clearly is higher in the proximal newt limb (Kumar et al., 2007a; Mercader et al., 2005). This might suggest a direct epigenetic inheritance of Prod 1 expression from adult limb cells to their blastemal derivatives. Nonetheless it is important to recognise that Meis activity is also regulated by nuclear localisation and this may be more significant than transcriptional expression in this context, thus permitting Meis to retain its upstream role (Mercader et al., 2005). It is also possible that Prod 1 signalling through the EGFR, or other as yet unidentified partners, might regulate Meis expression (Blassberg et al., 2011). The possibility that such reciprocal interactions might operate to regulate PD identity after amputation is now open to experiment. The ability to test promoter sequences for their activity on the PD axis should aid in the investigation of other transcription factors that may be implicated.

## 5 Conclusions

These data support the hypothesis that the Prod 1 promoter is regulated on the PD axis during limb regeneration in the axolotl. An important aspect of its elevated expression in proximal blastemal cells is apparently the action of Meis transcription factors on two consensus sites in the promoter, as evidenced by the effect of mutating these sites. These results provide an explanation for the identical phenotype resulting from ectopic expression of either Meis or Prod 1 in distal blastemal cells, that is the proximal displacement and contribution to more proximal structures in the regenerate. The regulatory interactions between these two may play a role in the events after amputation that confers PD identity on the limb blastema.

The following are the supplementary materials related to this article.

**Supplementary figure:**
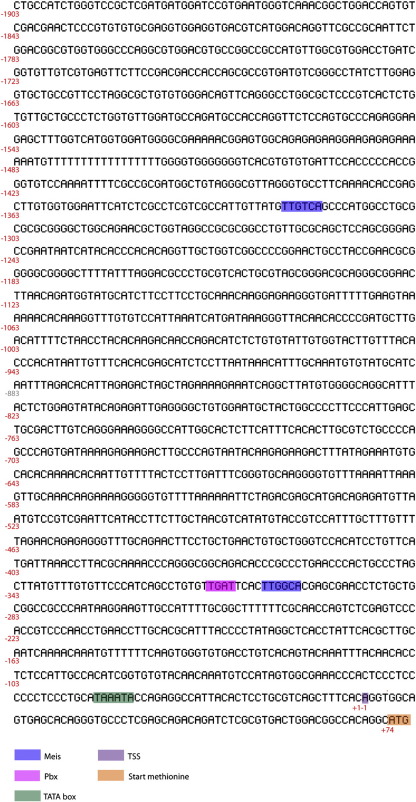
This shows the 1.9 kilobase of sequence analysed in the text. The binding sites for Meis and Pbx proteins are shown, as well as the TATA box, transcription start site (TSS) and start methionine codon.
